# Learning from aviation….Clinician Resource Management

**DOI:** 10.1308/rcsann.2024.0006

**Published:** 2024-02-01

**Authors:** B Rogers

**Figure:**
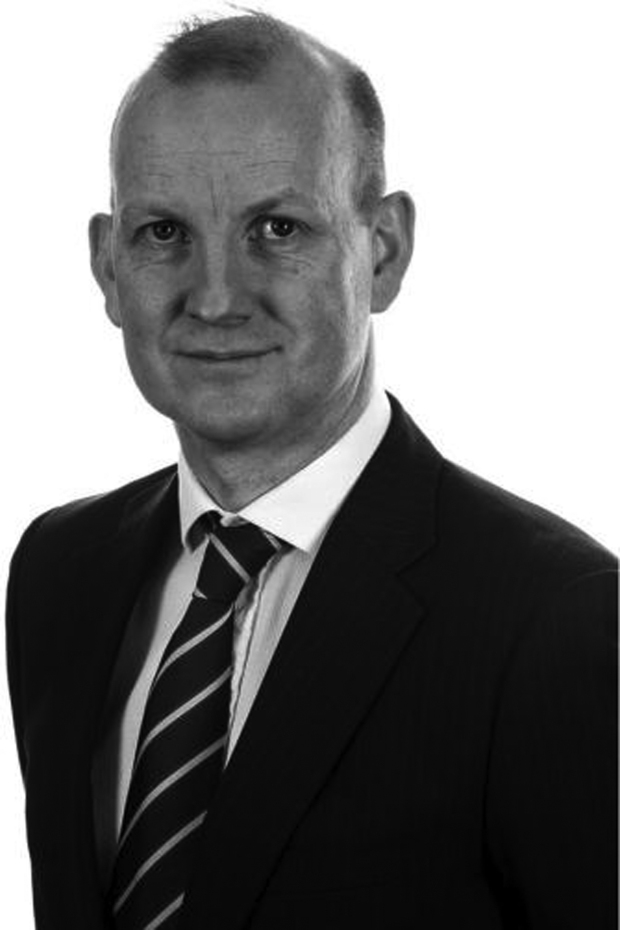
**B Rogers,** Editor-in-Chief *Annals* of the Royal College of Surgeons of England

This edition of the *Annals* has collated studies that consider some non-clinical aspects of surgical care that impact on patient outcomes. Topics of original research include clinician burnout, major trauma training and workforce planning. Such topics have obvious parallels with the aviation profession. In the 1970s, the introduction of cockpit voice recorders identified that ‘human factors’ contributed strongly to many aviation accidents. The majority of ‘pilot-related’ errors were, in fact, failures of interpersonal skills, communication, decision making and leadership rather than technical ability.

In 1978, a United Airlines DC8 plane crashed in Portland (US) after it ran out of fuel with the loss of ten lives. Analysis of cockpit voice recordings concluded that a contributory factor was the captain’s failure to accept input from other flight crew members, as well as the lack of assertiveness from those crew members themselves. As a result of this, the aviation authorities developed a comprehensive Crew Resource Management training which continues to this day. The parallels to surgical practice are clear.

It is clear that a surgeon’s technical skills alone are insufficient to ensure safety and best performance; accidents can occur for reasons other than inadequate surgical skills. It is apparent that clinicians need to learn more about how best to manage all the resources available to them in the operating theatre/ward/hospital, including other medical professionals, procedures, the interface with equipment interface, and themselves (i.e. recognising where they are most vulnerable and what their strengths are).

In this edition of the *Annals*, I am delighted to highlight the invited editorial by Prof Peter Brennan and Dr Steve Jarvis, leading experts on human factors, on the need and value of embedding human factors into surgical practice. I would recommend reading the 2023 Civil Aviation Authority *Flight Crew Human Factors Handbook,* edited and co-authored by Dr Steve Jarvis; it is enlightening for clinicians.^1^ Surgical practice can learn so much from the detailed and analytical way that aviation assesses and manages risk. As such, the *Annals* is keen to encourage analytical research on human factors within surgery and healthcare in general.

Pilots introduced cockpit voice recording over 50 years ago, with an associated improvement in safety. Is there an argument for introducing operating theatre voice recordings? In time, further research and data from the Confidential Reporting System in Surgery^2^ may enable the development of ‘clinician resource management’ along similar lines as our aviation colleagues.
